# Preparation and Characterization of Films Extruded of Polyethylene/Chitosan Modified with Poly(lactic acid)

**DOI:** 10.3390/ma8010137

**Published:** 2014-12-30

**Authors:** Jesús Manuel Quiroz-Castillo, Dora Evelia Rodríguez-Félix, Heriberto Grijalva-Monteverde, Lauren Lucero Lizárraga-Laborín, María Mónica Castillo-Ortega, Teresa del Castillo-Castro, Francisco Rodríguez-Félix, Pedro Jesús Herrera-Franco

**Affiliations:** 1 Departamento de Investigación en Polímeros y Materiales, Universidad de Sonora, C.P. 83000 Hermosillo, Sonora, Mexico; E-Mails: quiroz51@hotmail.com (J.M.Q.-C.); hgrijalv@guaymas.uson.mx (H.G.-M.); lizlauren2@hotmail.com (L.L.L.-L.); monicac@guaymas.uson.mx (M.M.C.-O.); terecat@polimeros.uson.mx (T.C.-C.); 2 Departamento de Ingeniería Ambiental Industrial, Universidad Estatal de Sonora, C.P. 83100 Hermosillo, Sonora, México; 3 Departamento de Investigación y Posgrado en Alimentos, Universidad de Sonora, C.P. 83000 Hermosillo, Sonora, Mexico; E-Mail: frodriguez@guayacan.uson.mx; 4 Unidad de Materiales, centro de Investigación Científica de Yucatán, C.P. 97200 Mérida, Yucatán, Mexico; E-Mail: pherrera@cicy.mx

**Keywords:** polyethylene, chitosan, poly(lactic acid), maleic anhydride, extrusion

## Abstract

The use of mixtures of synthetic and natural polymers is a potential option to reduce the pollution by plastic waste. In this work, the method for the chemical modification of chitosan with poly(lactic acid) was developed; then, the preparation of films of blends of polyethylene and chitosan-poly(lactic acid) produced by an extrusion method using polyethylene-graft maleic anhydride as a compatibilizer. It was possible to obtain films with a maximum content of 20 wt% and 30 wt%, chitosan, with and without compatibilizer, respectively. Scanning electron microscope (SEM) analysis showed a homogeneous surface on all films. The addition of the compatibilizer had a significant effect on the mechanical properties of the films, such as an increase in Young’s modulus and a decrease in the elongation at break; additionally, the compatibilizer promotes thermal degradation in a single step and gives the film a slight increase in thermal resistance. These results are attributed to an improved interaction in the interface of polyethylene and chitosan-poly(lactic acid), promoted by the compatibilizer.

## 1. Introduction

Polyethylene is the polyolefin most widely used worldwide. The range of applications includes food packaging containers or other types of disposable packaging and/or wrapping films for different substances or articles, all of great importance in everyday life. However, the enormous production and utilization of synthetic polymers has led to the accumulation of plastics, creating a serious source of pollution thereby affecting the environment [1,2].

Blends of synthetic and natural polymers can form a new class of materials with improved mechanical properties and biodegradability compared with those of single components. Moreover, these blends are an alternative that may contribute to the reduction of the environmental damage caused by the polymeric waste [3].

Chitosan is a biopolymer derived from the deacetylation of chitin, the main structural component of crustacean exoskeletons [4,5], thus, is one of the polysaccharides most commonly found in nature and its films have great potential to be used as packaging materials due to their antimicrobial activity, nontoxicity and biodegradability [6,7,8,9,10,11,12]. Chitosan is a rigid material which can be modified with biodegradable thermoplastics in order to improve its mechanical properties without affecting its biodegradability. Thus, chitosan can be modified with poly(lactic acid) (PLA) by means of an amidation reaction between the amino groups in the main chain of chitosan and terminal carboxil group in PLA.

Poly(lactic acid) is a highly versatile and biodegradable, aliphatic polyester derived from 100% renewable resources, such as corn and sugar beets. PLA offers great promise in a wide range of commodity applications [13]. PLA is a thermoplastic, high-strength, high-modulus polymer that can be made from annually renewable sources to yield articles for use in either the industrial packaging field or the biocompatible/bioabsorbable medical devices market. It is easily processed on standard plastics equipment to yield molded parts, film, or fibers [14]. In contrast, chitosan is difficult to process using these standard methods, such as extrusion molding, thus, the chemical modification of chitosan with PLA improved processing and allowed to obtain a material with intermediate properties.

Several studies have been reported related to the preparation of chitosan blends with thermoplastics, but the blends in those papers were prepared primarily by a solvent evaporation method [15,16] which could be associated to atmosphere pollution. The objective of this work is to obtain films based on blends of synthetic polymers with natural polymers, using extrusion which is a method, widely used industrially, with acceptable mechanical properties and environmentally-friendly as compared to conventional synthetic polymers. In this paper, we present the modification of chitosan with PLA followed by the preparation of films of blends of polyethylene and chitosan-PLA by the extrusion molding technique using polyethylene-graft-maleic anhydride as a compatibilizer [2] and its characterization by infrared spectroscopic, mechanical properties, scanning electron microscopy and thermogravimetric analysis.

## 2. Results and Discussion

### 2.1. Chemical Modification of Chitosan Using Poly(lactic acid)

The product obtained from the chemical modification reaction is a material with macroscopic physical characteristics very different in comparison with the individual polymers. This product is white colored and has a similar appearance to cotton. The yield percentage was about 95%. This product was analyzed by infrared spectroscopy to corroborate the successful modification of chitosan, and the morphology was studied by scanning electron microscope (SEM).

**Figure 1:**
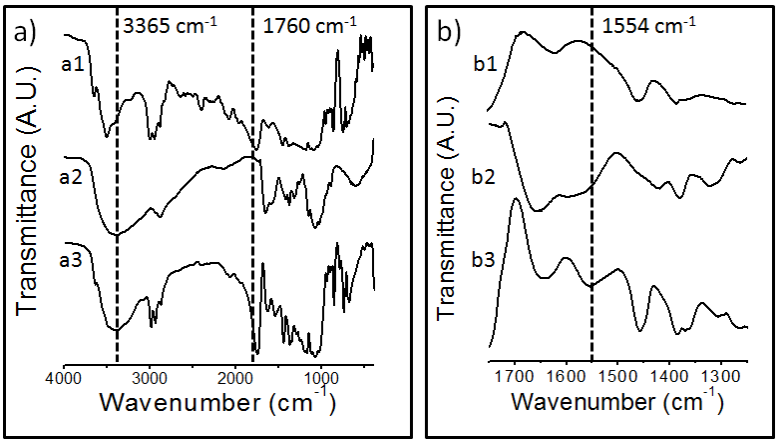
FTIR spectra of (**a**) full spectrum, (**b**) range of 1750–1250 cm^−1^: (1) chitosan, (2) PLA, (3) Ch-PLA.

**Figure 2:**
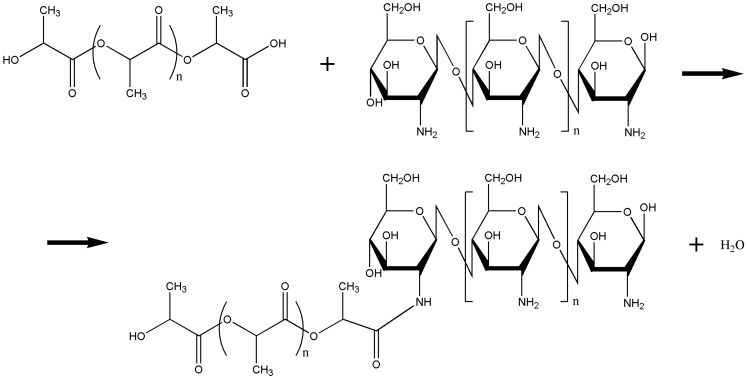
Scheme of the representation of chemical modification of chitosan with poly (lactic acid).

Fourier Transform Infrared Spectroscopy (FTIR) spectra are shown in Figure 1a,b. Spectrum (a1) shows the characteristic peaks of PLA, the carbonyl group at 1760 cm^−1^ and the peaks representing methyl stretch in a wave number between 2800 and 3000 cm^−1^, –CO– stretch in a wave number range from 1050 to 1250 cm^−1^ and –CC– stretch at 871 cm^−1^ [17]. The peak corresponding to the stretching of the OH group in the chitosan appears at 3365 cm^−1^ (a2). To achieve a more detailed analysis, a magnification of the spectral region of 1750–1250 cm^−1^ (Figure 1b) is presented. The spectrum of the modified chitosan (Ch-PLA) (a3) shows the spectral contributions of the PLA and chitosan, however, a new band at 1554 cm^−1^ appears. (b3), indicating the presence of a secondary amide (amide II) [18], the presence of this new band confirmed that an amidation reaction between chitosan and PLA was carried out. In Figure 2, the scheme of chemical modification of chitosan with poly (lactic acid) is shown, in this chemical modification a condensation reaction between amino groups of chitosan and terminal carboxyl groups of PLA, was carried out, with a release of water molecules as side product; and the formation of an amide bond, as was revealed by FTIR.

**Figure 3:**
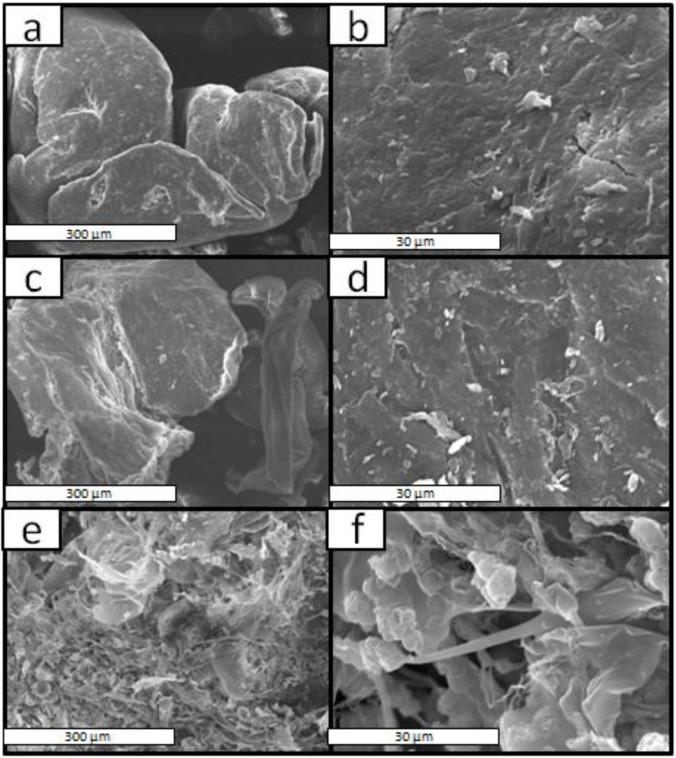
SEM micrographs of the surface of **(a**,**b**) PLA, (**c**,**d**) chitosan and (**e**,**f**) Ch-PLA, at (**a**,**c**,**e**) 200× and (**b**,**d**,**f**) 2000× magnification.

SEM micrographs for the PLA, chitosan and Ch-PLA at a magnification of 200× and 2000× are shown in Figure 3. In Figure 3a,c characteristic particles of PLA and chitosan are observed, respectively. While in micrographs of Ch-PLA (Figure 3e) the observed morphology is different from the individual polymers, and it is seen that both materials have undergone a change in their surface. By observing the surface of Ch-PLA at a higher magnification, Figure 3f, separate particles are not observed; furthermore, some holes may be observed, which are attributed to the physical appearance of the Ch-PLA, which is similar to that of cotton, because of this an uneven surface was detected.

### 2.2. Films of Polyethylene/Chitosan Modified with Poly(lactic acid) 

Films of Ch-PLA in a matrix of polyethylene were prepared. It was possible to extrude films with up to 20 wt% of Ch-PLA without compatibilizer in the mixture, while, the maximum amount of Ch-PLA in the films was 30% when 5% of polyethylene-graft-maleic anhydride (PEgMA) (compatibilizer) was added. Table 1 shows the compositions for each prepared mixture.

**Table 1:** Concentration of each component used in the preparation of blends.

Code	Composition in Films of PE and Ch-PLA		
	PE (wt%)	Ch-PLA (wt%)	PEgMA (wt%)
H1	95	5	0
H2	90	10	0
H3	85	15	0
H4	80	20	0
I1	90	5	5
I2	85	10	5
I3	80	15	5
I4	75	20	5
I5	70	25	5
I6	65	30	5

### 2.3. Characterization of Polyethylene/Chitosan Modified with Poly(lactic acid) Films

#### 2.3.1. Infrared Spectroscopy

**Figure 4:**
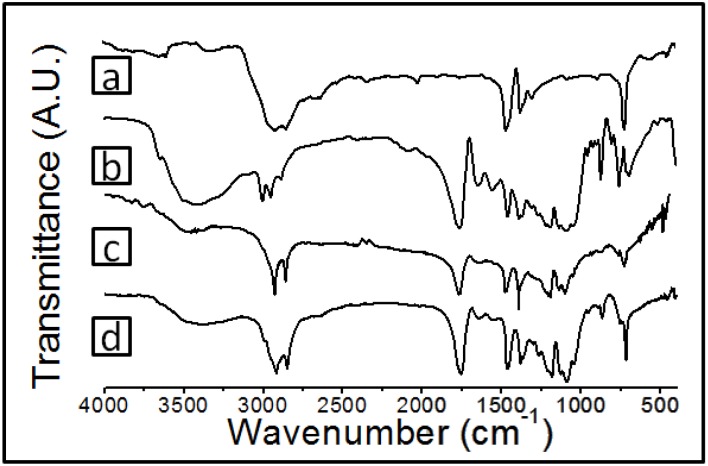
FTIR spectra of (**a**) PE, (**b**) Ch-PLA, (**c**) H3 film and (**d**) I3 film.

Figure 4 shows the FTIR spectra for the individual components and blends. Figure 4a shows the characteristic peaks of PE: (1) stretching the hydrocarbon peak at a wavenumber about 2800–3000 cm^−1^, (2) methylene scissoring peak motion at 1467 cm^−1^ and (3) methylene rocking band at 722 cm^−1^ [19]. In the case of Ch-PLA (Figure 4b ) the spectral contributions of the chitosan and the PLA are detected in addition to the new band due to the presence of the amide group at 1554 cm^−1^ .The spectra of the H4 and I6 films (Figure 4c,d) show the spectral contributions of the PE and Ch-PLA. No new band or peak displacement relative to the individual spectra of the components is observed. It was not possible to observe any interaction between PEgMA and Ch-PLA by this technique because the peak corresponding to the carbonyl group of the PEgMA appears at 1750 cm^−1^ and in the PLA at 1760 cm^−1^ [20], and the latter is of much greater intensity, preventing the observation of any possible displacement.

#### 2.3.2. Thermal Analysis 

**Figure 5:**
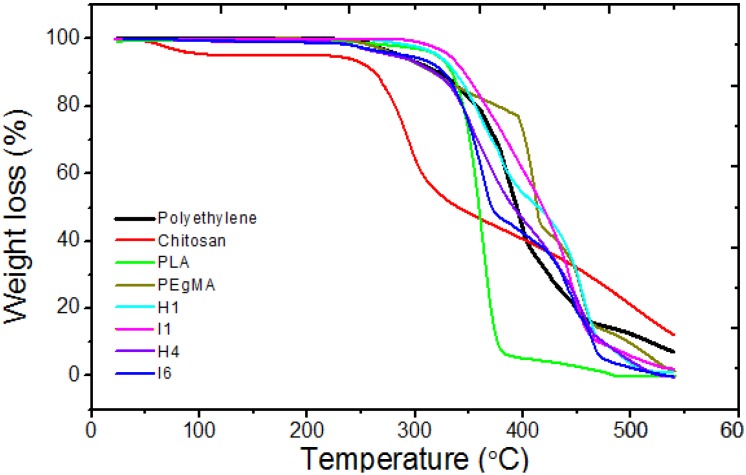
TGA curves for the polymeric materials and their blends.

Figure 5 shows the weight loss and heat flow for PE, PLA, chitosan, PEgMA and H1, H4, I1, I6 films as a function of temperature. 

In the case of PE/Ch-PLA film (H1, with 5 wt% of Ch-PLA, Figure 5e), the degradation is detected in two steps, the first step corresponds to the degradation of PE and chitosan, while the second step involves only PLA degradation which ends at about 400 °C, the thermal degradation of the film starts at a temperature of about 250 °C, due to the onset of thermal degradation of PE. 

By adding 5 wt% PEgMA, film I1, the material becomes more thermally stable and the weight loss is observed in a single step and with an onset at 300 °C (Figure 5f), this is an important indication of an improvement in the miscibility of the system, promoted by the presence of the compatibilizer. 

In films containing 20 wt% of Ch-PLA, but without compatibilizer, H4 film continues showing a thermal degradation in two stages. In the film I6, which contains compatibilizer, a thermal degradation in two steps was also observed, apparently, the compatibilizing effect offered by PEgMA 5 wt% is not enough to promote a weight loss in a single step. Importantly, none of the individual components and the films shows a weight loss when exposed to processing temperatures; therefore, there is no thermal degradation of the material during the extrusion process. 

Table 2 shows the thermal properties of PE and PE/Ch-PLA blends. No significant shift in the *T*_m_ of PE is noted with increasing Ch-PLA content, showing that the PE matrix and the Ch-PLA phase are only partially miscible. Pure PE films show a percentage of crystallinity of 36%, as Ch-PLA increases in the blends, the percentage of crystallinity diminishes, and this can be explained because, apparently, Ch-PLA inhibits the close packing of the PE chains. Similar behavior is reported in the literature for PE composites with chitosan [21] and wood flour [22].

**Table 2:** Thermal properties of PE and PE blends with Ch-PLA.

Film	*T*_m_ (°C)	Δ*H*_m_ (J/g)	Δ*H*_m_ (J/g)	*X*_c_ (%)
Polyethylene	106.65	107.58	298.86	36.00
H1	107.03	92.23	298.86	30.86
I1	107.98	65.64	298.86	21.96
H4	107.79	40.25	298.86	13.47
I6	108.28	39.76	298.86	13.30

#### 2.3.3. Analysis of Mechanical Properties 

**Figure 6:**
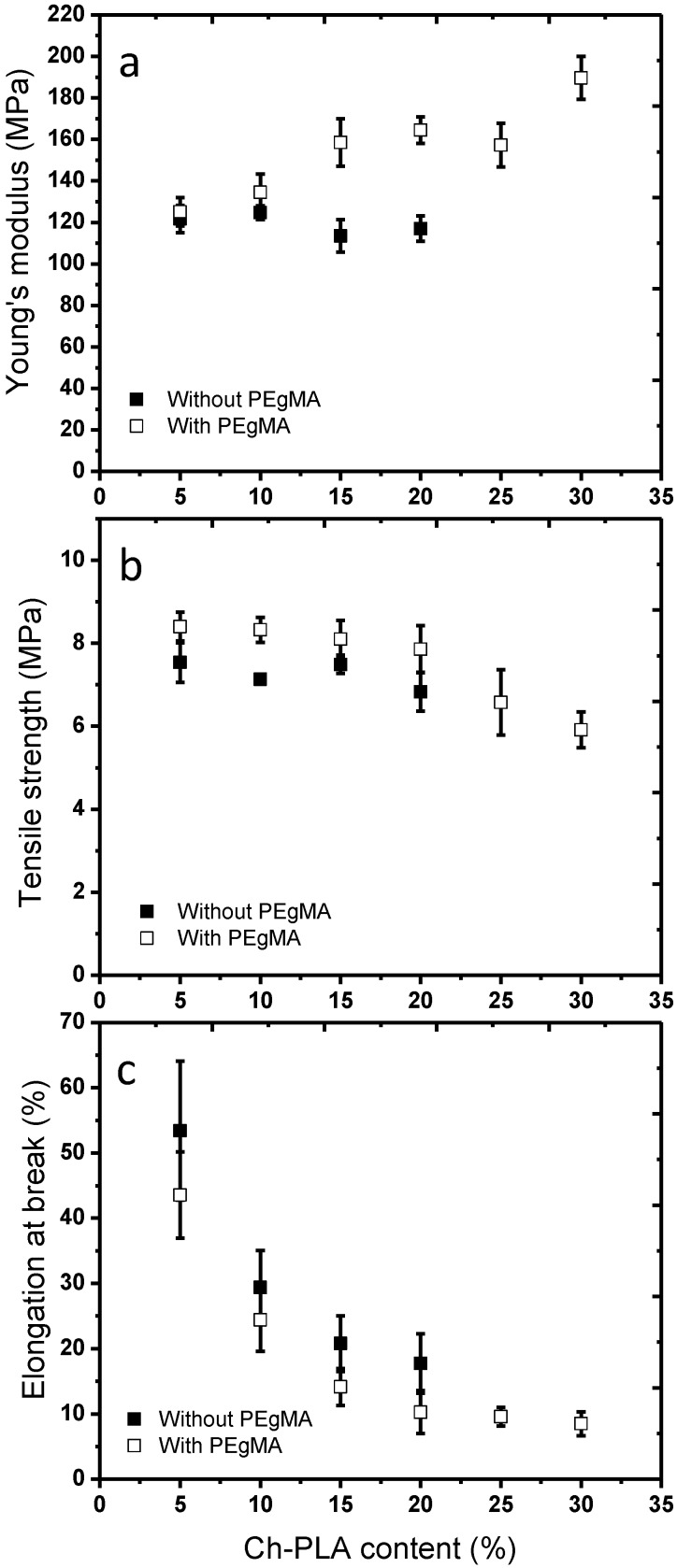
(**a**)Young’s modulus, (**b**) tensile strength and (**c**) elongation at break, in films of PE/Ch-PLA as a function to Ch-PLA content.

Figure 6 shows the Young’s modulus, tensile strength and elongation at break of the extruded film of PE and Ch-PLA as a function of the concentration of Ch-PLA. In the case of the films without compatibilizer, no significant change is observed in Young’s modulus with increasing the concentration of Ch-PLA. However, for the compatibilized films, a significant increase was detected in Young’s modulus, becoming 50% higher when a 30 wt% of Ch-PLA was added in the blend; because the Ch-PLA is a more rigid material than the PE, a similar behavior was observed in our previous paper where films of PE and chitosan were analyzed [2].

The tensile strength of the films of PE with Ch-PLA remained constant when the amount of Ch-PLA in the film was increased to a 20 wt%, regardless of the presence of PEgMA compatibilizer. Meanwhile, in films containing 25 wt% and 30 wt% of Ch-PLA, a decrease in tensile strength was observed. Again, a similar behavior was observed for the films studied in our previous paper [2]. The decrease of the tensile strength with increasing content of Ch-PLA was attributed to an increase in the number of imperfections in the material. 

Moreover, a decrease in elongation at break of the film with an increase of the content of Ch-PLA was observed. This behavior was attributed to the addition of a more rigid material compared to PE. The addition of PEgMA in the blend of PE and Ch-PLA, caused a more pronounced decrease in the elongation at break, in all compositions, indicating a stronger interaction between the polymer chains of PE and Ch-PLA in the interface.

#### 2.3.4. Scanning Electron Microscopy of Film Surfaces

**Figure 7:**
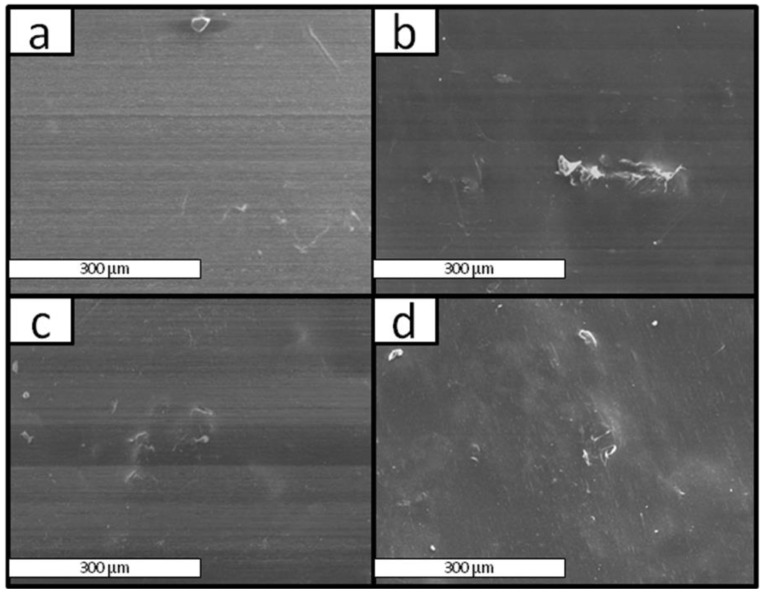
SEM micrographs of the surface of films (**a**) H1, (**b**) I1, (**c**) H4 and (**d**) I6.

Figure 7 shows the SEM micrographs of the surface of films of PE/Ch-PLA. The films of PE with 5 wt% of Ch-PLA showed a homogeneous surface regardless of the presence of PEgMA, as shown in Figure 7a,b. Moreover, the surface remains uniform regardless of the content of Ch-PLA in the film and the presence of PEgMA, even when the concentration of Ch-PLA is 20 wt% for films without compatibilizer and 30 wt% for the compatibilized films (Figure 7c,d, respectively). Then, it is not possible to determine the effect of the PEgMA compatibilizer for films of PE/Ch-PLA through these morphological results.

## 3. Experimental Section 

### 3.1. Materials

Commercial grade low density polyethylene (PE) (melt flow rate 2.0 g/10 min with 2.16 kg standard die at 190 °C), was obtained from Qatar Petrochemical Company (QAPCO, Doha, Qatar). Poly (lactic acid) (PLA) 2002d, (molecular weight of 192,610 g/mol) for extrusion and thermoforming, was obtained from the company Natureworks (Blair, NE, USA). Chitosan of medium molecular weight (molecular weight of 190,000–310,000 Da, and 75%–85% deacetylated), polyethylene-graft-maleic anhydride (PEgMA, with 3.5% maleic anhydride), acetic acid, tetrahydrofuran (THF), carbodiimide hydrochloride, *N*-(3-dimethylaminopropyl) (EDC) and ethylenediamine tetraacetic acid (EDTA) were obtained from Sigma-Aldrich (St. Louis, MO, USA). PE and PLA were milled in a Thomas Wiley Laboratory Mill (Swedesboro, NJ, USA), Model 4, down to a particle diameter of approximately 1 mm, and chitosan was dried at 110 °C for 24 h before usage. All other reagents were used as received.

### 3.2. Modification of Chitosan with Poly(lactic acid)

The modification of chitosan was carried out by an amidation reaction of the amino groups in the glucosamine units of the biopolymer and the terminal carboxyl groups in the chains of PLA. Initially one gram of chitosan was dissolved in 100 mL of dilute aqueous solution of acetic acid 1% (*m*/*v*); after this, 5 g of PLA dissolved in 200 mL of THF was added, next 0.39 g of EDC were added as a catalyst [23,24,25] and the mixture was stirred for 3 h. 

Purification of the modified chitosan was performed through a process of dialysis in deionized water for 48 h with water changes every 4 h. After this time, the solution in the dialysis bags was recovered and was further freeze-dried during 48 h in a Labconco Freezone 4.5 unit under a vacuum atmosphere of 0.5 mBar at −46 °C in the collector.

### 3.3. Preparation of Extruded Films of Polyethylene and Chitosan Modified

Films of PE and chitosan-modified with PLA (Ch-PLA) and films using polyethylene-graft-maleic anhydride (PEgMA) as a compatibilizer for the blend were prepared. The polymer blends of PE with Ch-PLA were subjected to mechanical agitation for 30 min. until a homogeneous mixture was obtained, in the compatibilized blends, PEgMA was added in this step.

The blends were then extruded in an Atlas laboratory mixer-extruder (Plantation, FL, USA), with a speed of 40 rpm. Temperatures were controlled at 145 and 155 °C for the rotor and the head respectively. 

### 3.4. Infrared Spectroscopic Analysis 

FT-IR spectroscopy analysis was performed with an FTIR Perkin-Elmer 1600 spectrophotometer (Waltham, MA, USA). Spectrum scan was performed from 4000 to 400 cm^−1^. An average of 32 scans was recorded. Approximately 5 mg of sample was mixed with KBr directly to form a pellet; the measurement was performed in the transmittance mode. 

### 3.5. Thermal Analysis 

The thermal behavior of the materials was studied by thermogravimetric analysis (TGA) and differential scanning calorimetry (DSC) using a simultaneous TGA and DSC equipment brand TA Instruments (New Castle, DE, USA), model SDT 2960; about 6 mg of sample were placed in the sample holder of alumina, and was subjected to a temperature increase at a rate of 10 °C min^−1^ from room temperature to 550 °C, in an air flow of 23 mL min^−1^.

Δ*H*_m_ was directly measured from the DSC curves, the percent of crystallinity (*X*_c_) for the PE films and the blends was calculated as:

*X*_c_ = 100 Δ*H*_m_/Δ*H*_m_ (1)

where Δ*H*_m_ is the melting enthalpy for crystalline polyethylene, *i.e.*, 298.86 J/g [26].

### 3.6. Analysis of Mechanical Properties 

The mechanical properties of the films were measured for tensile loading using a universal machine United SSTM-5kN (Huntington Beach, CA, USA) with head displacement rate of 10 mm min^−1^. At least eight specimens (dimensions: 5 mm × 50 mm) of each film were tested and the average values are reported. The thickness of the films were measured using a Mitutoyo micrometer and were approximately 1 mm.

### 3.7. Scanning Electron Microscopy 

The surface characteristics of the materials were first studied for the unmixed materials and one representative specimen of each composition of the prepared films was selected to study the surface morphology. This was performed using a scanning electron microscope (SEM) JEOL 5410LV (Tokyo, Japan), equipped with a system INCA dispersive X-ray detector (Oxford Instruments, Austin, TX, USA), operated at a voltage of 20 kV. Samples were coated with gold before being observed under a high vacuum using the secondary electron detector.

## 4. Conclusions 

Chitosan was successfully modified with poly (lactic acid) as was shown by the FT-IR, and moreover, the yield percentage was about 95%. 

Processing of films from blends of PE and Ch-PLA by extrusion was possible up to a maximum content of 20 wt% of Ch-PLA for films without compatibilizer and 30 wt% for the films with compatibilizer, and no phase separation was observed in the films with different compositions. 

The addition of the PEgMA as compatibilizer had a significant effect on the mechanical properties of the films of PE with Ch-PLA, as an increase in Young’s modulus and decrease in elongation at break; the tensile strength remained unchanged for Ch-PLA contents up to 20 wt%. It was also observed that the films with PEgMA showed a thermal degradation in a single step resulting in a greater compatibility between the components of the polymer blends and a slight increase in the thermal resistance. The changes in the properties of the films are interpreted to be the result of an improved interaction of PE and Ch-PLA in the interface, promoted by the presence of the compatibilizer.

It was possible to obtain films of synthetic polymer, PE with natural polymers, Ch-PLA, using a method very used industrially, with acceptable properties and more friendly to the environment compared to conventional synthetic polymers.

## References

[1] Arutchelvi J., Sudhakar M., Arkatkar A., Doble M., Bhaduri S., Veera Uppara P. (2008). Biodegradation of polyethylene and polypropylene. Indian J. Biotechnol..

[2] Quiroz-Castillo J.M., Rodríguez-Félix D.E., Grijalva-Monteverde H., del Castillo-Castro T., Plascencia-Jatomea M., Rodríguez-Félix F., Herrera-Franco P.J. (2014). Preparation of extruded polyethylene/chitosan blends compatibilized with polyethylene-graft-maleic anhydride. J. Carbohydr. Polym..

[3] Sionkowska A. (2011). Current research on the blends of natural and synthetic polymers as new biomaterials: Review. Prog. Polym. Sci..

[4] Cai K., Yao K., Cui Y., Lin S., Yang Z., Li X., Xie H., Qing T., Luo J. (2002). Surface modification of poly (d,l-lactic acid) with chitosan and its effects on the culture of osteoblasts *in vitro*. J. Biomed. Mater. Res..

[5] Dang Q.F., Zou S.H., Chen X.G., Liu C.S., Li J.J., Zhou X., Liu Y., Cheng X.J. (2012). Characterizations of chitosan-based highly porous hydrogel-the effects of the solvent. J. Appl. Polym. Sci..

[6] Harish-Prashanth K.V., Tharanathan R.N. (2007). Chitin/chitosan: Modifications and their unlimited application potential—An overview. Trends Food Sci. Technol..

[7] Li J., Zivanovic S., Davidson P.M., Kit K. (2010). Characterization and comparison of chitosan/PVP and chitosan/PEO blend films. Carbohydr. Polym..

[8] Nair L.S., Laurencin C.T. (2007). Biodegradable polymers as biomaterials. Prog. Polym. Sci..

[9] Pillai C.K.S., Paul W., Sharma C.P. (2009). Chitin and chitosan polymers: Chemistry, solubility and fiber formation. Prog. Polym. Sci..

[10] Shih C.M., Shieh Y.T., Twu Y.K. (2009). Preparation and characterization of cellulose/chitosan blend films. Carbohydr. Polym..

[11] Sindhu M., Abraham T.E. (2008). Characterisation of ferulic acid incorporated starch-chitosan blend films. Food Hydrocoll..

[12] Zhong C., Wu J., Reinhart-King C.A., Chu C.C. (2010). Synthesis, characterization and cytotoxicity of photo-crosslinked maleic chitosan-polyethylene glycoldiacrylate hybrid hydrogels. Acta Biomater..

[13] Drumright R.E., Gruber P.R., Henton D.E. (2000). Polylactic acid technology. Adv. Mater..

[14] Garlotta D. (2002). A literature review of poly(lactic acid). J. Polym. Environ..

[15] Feng H., Dong C.M. (2006). Preparation, characterization, and self-assembled properties of biodegradable chitosan-poly(l-lactide) hybrid amphiphiles. Biomacromolecules.

[16] Kuo P.C., Sahu D., Yu H.H. (2006). Properties and biodegradability of chitosan/nylon 11 blending films. Polym. Degrad. Stab..

[17] Copinet A., Bertrand C., Govindin S., Coma V., Couturier Y. (2004). Effects of ultraviolet light (315 nm), temperature and relative humidity on the degradation of polylactic acid plastic films. Chemosphere.

[18] Larez-Velasquez C., Rivas A., Velasquez W., Bahsas A. (2007). Amidación del quitosano con cloruro de oleoilo. Rev. Iberoam. Polim..

[19] Del Castillo-Castro T., Castillo-Ortega M.M., Herrera-Franco P.J., Rodriguez-Felix D.E. (2011). Compatibilization of polyethylene/polyaniline blends with polyethylene-graft-maleic anhydride. J. Appli. Polym. Sci..

[20] Singh G., Bhunia H., Rajor A., Choudhary V. (2011). Thermal properties and degradation characteristics of polylactide, linear low density polyethylene, and their Blends. Polym. Bull..

[21] Mir S., Yasin T., Halley P.J., Siddiqi H.M., Nicholson T. (2011). Thermal, rheological, mechanical and morphological behavior of HDPE/chitosan blend. Carbohydr. Polym..

[22] Magnus B., Kristiina O. (2006). The use of silane technology in crosslinking polyethylene/wood flour composites. Compos. A Appl. Sci. Manuf..

[23] Duhamel J., Kanagalingam S., O’Brien T.J., Ingratta M. (2003). Side-chain dynamics of an alpha-helical polypeptide monitored by fluorescence. J. Am. Chem. Soc..

[24] Telmo H., Prazeres J., Duhamel J. (2005). Characterization of the aggregates made by short poly(ethylene oxide) chains labeled at one end with pyrene. Macromolecules.

[25] Zhu A., Zhang M., Wu J., Shen J. (2002). Covalent immobilization of chitosan/heparin complex with a photosensitive hetero-bifunctional crosslinking reagent on PLA surface. Biomaterials.

[26] Sutivisedsak N., Cheng H.N., Dowd M.K., Selling G.W., Biswas A. (2012). Evaluation of cotton byproducts as fillers for poly(lactic acid) and low density polyethylene. Ind. Crops Prod..

